# Prognostic value of coagulation markers in patients with colorectal cancer: A prospective study

**DOI:** 10.1002/hsr2.1553

**Published:** 2024-01-31

**Authors:** Wenxin Chen, Yueying Li, Weifeng Wang, Yingjun Xue, Jianxin Qian, Weiwei Liu, Xiaobo Hu

**Affiliations:** ^1^ Department of Laboratory Medicine, Longhua Hospital Shanghai University of Traditional Chinese Medicine Shanghai China; ^2^ Department of Laboratory Medicine and Central Laboratory, Shanghai Tenth People's Hospital Tongji University School of Medicine Shanghai China; ^3^ Department of Oncology Longhua Hospital Affiliated to Shanghai University of Traditional Chinese Medicine Shanghai China; ^4^ General Office Shanghai Center for Clinical Laboratory Shanghai China

**Keywords:** coagulation, colorectal cancer, diagnosis, nomogram, prognosis

## Abstract

**Background and Aims:**

The occurrence, growth, and metastasis of colorectal cancer (CRC) are connected to the hypercoagulable state of blood (CRC). This study aimed to identify significant coagulation factors to predict metastasis and prognosis of CRC.

**Methods:**

Thrombomodulin (TM), thrombin‐antithrombin complex (TAT), α2‐plasmininhibitor‐plasmin complex (PIC), and tissue plasminogen activator‐inhibitor complex (t‐PAIC) were detected by chemiluminescence immunoassay using Sysmex HISCL5000 automated analyzers. The Sysmex CS 5100 automatic blood coagulation analyzer was used to detect d‐dimer (DD), fibrin degradation product (FDP), prothrombin time (PT), thrombin time (TT), international normalized ratio (INR), fibrinogen (Fbg), and activated partial thromboplastin time (APTT). Area under the curve (AUC) and the receiver operating characteristic curve (ROC) were used to assess the diagnostic efficacy of markers. Kaplan–Meier analysis was used to calculate survival probabilities. Independent prognostic factors and the nomogram were developed using single‐factor and multifactor cox regression analysis model.

**Results:**

The following indicators (TM, TAT, PIC, t‐PAIC, DD, FDP, PT, INR, APTT, and Fbg) were markedly higher in CRC patients than in healthy controls, and they were higher in the metastasis (M) group than in the nonmetastasis (NM) group. The combination “TAT + PIC + DD + FDP + Fbg” can distinguish M from NM with exceptional sensitivity and specificity. Patients with CRC who had high levels of TAT, PIC, DD, FDP, Fbg, TM, tPAIC, PT, and INR had significantly shorter survival.

**Conclusion:**

The prognosis of CRC patients can be predicted by coagulation indicators. The independent predictive variables for overall survival were found to be TM and DD. To forecast CRC patient survival, a nomogram was created.

## 1 INTRODUCTION

The third most prevalent neoplastic disease worldwide and a major factor in tumor‐related death is colorectal cancer (CRC).^1^, ^2^ In most cases, CRC cannot be detected in the early phases, and symptoms appear when the patient has reached an advanced or incurable stage.^3^ Cancer patients may experience a risk of bleeding due to the procoagulant active chemicals that cancer cells release.^4^ Coagulation disorders may be the first symptom of malignancy, and the majority of cancer patients die from thrombotic and hemorrhagic consequences. Patients with metastatic cancer are more likely than those without it to develop venous thrombosis.^5^ Tumor growth is associated with angiogenesis and end‐organ damage, and coagulation activation may be observed in patients with cancer.^6^ Anticancer drugs can also promote the development of coagulation issues. Some tumors release procoagulant chemicals and trigger the coagulation cascade. These chemicals cause an inflammatory reaction, and further inflammation induces the release of procoagulant chemicals from tumor cells.^7^

Functional indicators of the fibrinolytic system include fibrin degradation product (FDP) and d‐dimer (DD), and their levels considerably rise in the presence of elevated fibrinolytic activity, which may signal blood coagulation system activity.^8^, ^9^ In cancer patients, metastasis and a poor prognosis are associated with high levels of DD.^10^, ^11^, ^12^, ^13^ However, several factors can interfere with the detection of DD in the laboratory, including rheumatoid factor, heterophile antibodies, and immune complexes.^14^ The development of detection assays led to the use of coagulation and fibrinolysis biomarkers such as thrombin‐antithrombin complex (TAT), α2‐plasmininhibitor‐plasmin complex (PIC), thrombomodulin (TM), and tissue plasminogen activator‐inhibitor complex (t‐PAIC) in the clinical laboratory with a new testing method, the chemiluminescence immunoassay.^15^ TAT is created when thrombin and antithrombin combine, its production signifies the production of thrombin and, consequently, the activation of the coagulation mechanism^16^, ^17^ An early aberrant coagulation function in the body is suggested by increased levels of TAT.^18^ When the vascular endothelium is damaged, TM levels are abnormally upregulated, which is helpful to determine the severity of vascular injury.^19^ By forming an extremely strong bond with thrombin and changing its procoagulant substrate specificity to an anticoagulant, TM likewise functions as an anticoagulant. Plasmin and α2‐antiplasmin combine to create PIC in a 1:1 ratio. The presence of PIC suggests that fibrinolytic enzymes have been activated.^20^, ^21^ Tissue plasminogen activator (t‐PA) and plasminogen activator inhibitor‐1 (PAI‐1) combine in a 1:1 ratio to generate T‐PAIC. Vascular endothelial cells, which are primarily responsible for producing T‐PA, convert plasminogen into fibrinolytic enzyme. T‐PAIC is a biomarker of the fibrinolytic system's activation as well as damage to the vascular endothelium.^22^

This study's objective was to investigate into changes in patients with CRC in terms of TAT, PIC, TM, and t‐PAIC, as well as fibrinolysis biomarkers and conventional clotting biomarkers in CRC patients with metastasis. To predict overall survival (OS) in CRC patients, we also constructed a nomogram incorporating independent prognostic markers.

## 2 MATERIALS AND METHODS

### 2.1 Patients and healthy controls (HCs)

This study included 167 CRC patients who received a CRC diagnosis between January 2019 and July 2019 at the Department of Oncology Shanghai Tenth People's Hospital (Shanghai, China). The pursuing inclusion standards were used: (a) individuals with colorectal cancer whose histological confirmation was obtained from surgical or endoscopic specimens, (b) those who did not take any anticoagulants, (c) all chosen patients who did not get chemotherapy within a week of the study. The following conditions precluded patients: (a) infections, (b) coagulation abnormality, (c) using anticoagulant medications (d) various cancerous conditions. Characteristics including sex, age, pathology, stage, tobacco habits, and underlying conditions (hypertension, coronary heart disease, diabetes) of all patients were recorded. The clinical features of patients were also collected. Patients were classified into a metastasis group (M, *n* = 116) and a nonmetastasis group (NM, *n* = 51) according to metastatic lesion identification based on based on a laparotomy, image‐guided biopsy, or imaging examination. In addition, 83 HCs without hypertension, diabetes, or coagulation‐related diseases who went to the hospital for a physical checkup were chosen as the HC group. Up until June 2022, phone calls and hospital re‐examinations were used to follow up with every patient. Once every 3 months, a follow‐up was conducted to document the patients’ health state or eventual demise. The Human Ethics Review Committee of Shanghai Tenth People's Hospital gave its approval to the study protocol (File no: 2020‐KN155‐01). All methods were carried out with the relevant guidelines and regulations.

### 2.2 Clinical laboratory detection

All patients were analyzed for TM, TAT, PIC, t‐PAIC, traditional coagulation biomarkers, tumor biomarkers, and routine biochemical indicators. TAT, PIC, TM, and t‐PAIC were determined using the appropriate assay kits and a Sysmex‐HISCL5000 automated analyzer (HISCL‐5000i, Sysmex). Traditional coagulation biomarkers PT, APTT, TT, Fbg, FDP, DD, antithrombin (AT), and international normalized ratio (INR) were detected with a Sysmex CS5100 coagulation analyzer (Sysmex) using the corresponding assay kits. Tumor biomarkers carbohydrate antigen (CA) 199, cytokeratin fragment 19 (CYFRA211), CA724, neuron‐specific enolase (NSE), alpha fetoprotein (AFP), CA125, CA153, carcinoembryonic antigen (CEA), and squamous cell carcinoma antigen (SCC) were measured with the Roche cobas e801 (Roche, Switzerland) using the corresponding assay kits. Routine biochemical indicators albumin (ALB), aspartate aminotransferase (AST), and gamma‐glutamyl transpeptidase (GGT) were measured with a Beckman Coulter AU5800 Chemistry Analyzer (Beckman Coulter, Inc., Brea, CA, USA) using the corresponding assay kits.

The systemic immune‐inflammation index was calculated using the absolute platelet (P), neutrophil (N), and lymphocyte (L) counts (SII = P × [N/L]). Platelets, neutrophils, and lymphocytes were measured using a Sysmex XN9000 (Sysmex) with the corresponding kits.

### 2.3 Statistical analysis

Statistical analysis was performed using SPSS version 26 (IBM Corp.) and GraphPad Prism version 9.0 (GraphPad Software, Inc.). Nomograms were generated using R software version 3.6.2. For all tests and analyses, *p* values less than 0.05 were regarded as statistically significant for all tests and analyses. Data were evaluated for normalcy. Continuous data with a normally distribution are expressed as mean and standard deviation (SD); Otherwise, quartile ranges from the lower quartile Q1 to the upper quartile Q3 and the median (M) were employed. Using a one‐way analysis of variance and a two‐tailed unpaired *t* test, significant differences among the groups were discovered (analysis of variance). The maximum value of the Youden index served as the cutoff value for the receiver operating characteristic (ROC) curves that were used to assess the diagnostic effectiveness of the markers. Single indicator‐based ROC curves and column graphs were calculated and plotted using GraphPad. ROC curves for combined indicators were calculated using SPSS and plotted using GraphPad. The distribution of OS was depicted using Kaplan–Meier survival curves, and the log‐rank test was used to identify any differences that were statistically significant. The simultaneous impact of prognostic variables on survival were calculated using a Cox proportional hazards model. The multivariate analysis included every variable that was statistically significant in the univariate analysis. All independently related prognostic variables for OS were integrated into a predictive nomogram.

### 2.4 The specific model building

Area under the curve (AUC) and the ROC curve were used to assess the diagnostic efficacy of markers. Kaplan–Meier analysis was used to calculate survival probabilities. Independent prognostic factors and the nomogram were developed using single‐factor and multifactor cox regression analysis model. Because the nomogram is a graphical and quantitative tool for predicting classification, it allows multiple variables to be considered simultaneously, including an established classification system that is more efficient. First, the univariate and multivariate a Cox proportional hazards model were used to identify risk factors related to outcomes. Variables were included in the second step of the multivariable Cox proportional hazards model with backward selection (likelihood‐ratio test) if they were found significantly associated with our outcomes in the first step of univariate Cox proportional hazards model. The above analyses were performed using SPSS version 26. A *p* < 0.05 was indicated a statistically significant difference. Second, a novel prognostic nomogram based on coagulation markers was established for predicting the prognosis of CRC patients using the R software version 3.6.2.

## 3 RESULTS

### 3.1 Baseline characteristics

The study consisted of 167 CRC patients in total. Retrospective analysis was done on the patient data. Table 1 provides an overview of the participant baseline characteristics. The follow‐up was censored to June 2022, and 48 patients were confirmed dead, whereas 45 were still alive by the last follow‐up. The remaining 74 patients could not be reached for various reasons.

**Table 1:** Baseline characteristics.

	Entire cohort	M	NM	*p* value
*n*	167	116 (69.4)	51 (30.5)	
Age	64.11 ± 11.10	63.97 ± 11.72	64.45 ± 9.62	
Male/female	96 (57.49%)/71 (42.51%)	65 (56.03%)/51 (43.97%)	31 (60.78%)/20 (39.22%)	
Site				
Colon	99 (59.2%)	71 (61.2%)	28 (54.9%)	
Rectum	68 (40.7%)	45 (38.7%)	23 (45.1%)	
Diabetes				
Yes	34 (20.3%)	24 (20.6%)	10 (19.6%)	
No	133 (79.6%)	92 (79.3%)	41 (80.3%)	
Hyertension				
Yes	49 (29.3%)	36 (31%)	13 (25.4%)	
No	118 (70.6%)	80 (68.9%)	38 (74.5%)	
SII	412.05 (258.5, 639.28)	425.83 (265.38, 757.05)	354.35 (234.04, 536.15)	0.04*
TM (TU/mL)	9.38 ± 2.47	9.35 ± 2.32	9.42 ± 2.81	0.87
TAT (μg/L)	1.9 (1.1, 4.05)	2.3 (1.4, 5.63)	1.1 (0.8, 2)	<0.001*
PIC (mg/L)	0.81 (0.6, 1.22)	0.944 (0.66, 1.45)	0.63 (0.48, 0.85)	<0.001*
TAT/PIC	2.27 (1.58, 4.01)	2.37 (1.74, 4.12)	2 (1.37, 2.99)	0.30
tPAIC (μg/L)	8.03 ± 3.32	8.59 ± 3.45	6.74 ± 2.60	0.003*
AT (%)	92.09 ± 12.78	92.21 ± 12.91	91.82 ± 12.6	0.86
FDP (mg/L)	2.7 (1.48, 5.4)	3.5 (2.2, 7.7)	1.8 (0.9,3.1)	<0.001*
Fbg (g/L)	3.60 ± 1.37	3.94 ± 1.42	2.83 ± 0.86	<0.001*
PT (s)	11.28 ± 0.79	11.36 ± 0.77	11.09 ± 0.79	0.04*
INR	0.98 ± 0.07	0.99 ± 0.07	0.96 ± 0.07	0.04*
APTT (s)	27.22 ± 2.12	27.13 ± 2.22	27.43 ± 1.88	0.40
TT (s)	16.79 ± 0.98	16.64 ± 0.96	17.14 ± 0.94	0.002*
DD (mg/L)	0.68 (0.28, 1.64)	0.98 (0.4, 2.20)	0.29 (0.19, 0.69)	<0.001*
ALB (g/L)	39.43 ± 3.89	39.15 ± 4.03	40.10 ± 3.49	0.13
AST (U/L)	22 (19, 30)	24 (19, 30)	20 (17, 25)	0.09
GGT (U/L)	31.5 (19, 65)	38 (21, 84.25)	23 (17, 36)	0.005*
CA199 (U/mL)	18.7 (9.64, 45.05)	23.07 (11.58, 93.54)	10.38 (7.21, 25.57)	<0.001*
CYFRA211 (ng/mL)	3.91 (2.31, 7.81)	4.52 (2.69, 9.89)	2.51 (1.96, 4.24)	<0.001*
CA724 (U/mL)	3.83 (2.1, 11.84)	5.34 (2.44, 15.82)	2.35 (1.2, 4.71)	<0.001*
NSE (ng/mL)	13.29 (11.39, 16.68)	13.66 (11.44, 18.39)	12.55 (11.2, 14.97)	0.04*
AFP (ng/mL)	3.22 (2.23, 4.39)	3.38 (2.37, 4.67)	2.73 (1.90, 3.72)	0.04*
CA125 (U/mL)	13.8 (8.83, 22.48)	17.9 (9.1, 32.4)	10.8 (8.15, 15)	<0.001*
CA153 (U/mL)	10.6 (7.4, 14.7)	12 (8.3, 14.8)	7.6 (6.1, 11.45)	<0.001*
CEA (ng/mL)	4.8 (2.4, 41)	8.6 (3.1, 120.7)	2.6 (1.75, 3.95)	<0.001*
SCC (ng/mL)	0.8 (0.7, 1.2)	0.9 (0.7, 1.2)	0.8 (0.65, 1.45)	0.66

Abbreviations: APTT, activated partial thromboplastin time; AT, antithrombin. DD, d‐dimer; Fbg, fibrinogen; FDP, fibrin degradation product; HC, healthy control; INR, international normalized ratio; M, metastasis group; NM, nonmetastasis group; PIC, α2‐plasmininhibitor‐plasmin complex; PT, prothrombin time; TAT, thrombin‐antithrombin complex; TM, thrombomodulin; t‐PAIC, tissue plasminogen activator‐inhibitor complex; TT, thrombin time.

* *p* < 0.05 considered statistically significant.

**Figure 1:**
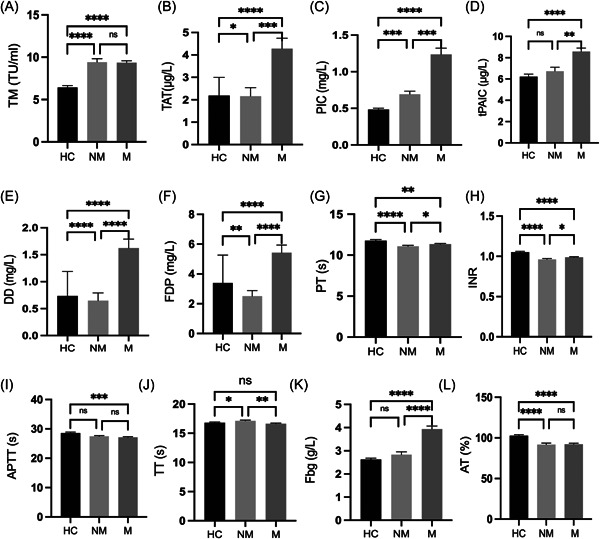
Differences in coagulation biomarkers between the healthy control, metastasis, and nonmetastasis groups. *p < 0.05, **p < 0.01, ***p < 0.001, ****p < 0.0001. APTT, activated partial thromboplastin time; AT, antithrombin; DD, d‐dimer; Fbg, fibrinogen; FDP, fibrin degradation product; HC, healthy control; INR, international normalized ratio; M, metastasis group; NM, nonmetastasis group; PIC, α2‐plasmininhibitor‐plasmin complex; PT, prothrombin time; TAT, thrombin‐antithrombin complex; TM, thrombomodulin; t‐PAIC, tissue plasminogen activator‐inhibitor complex; TT, thrombin time.

**Figure 2:**
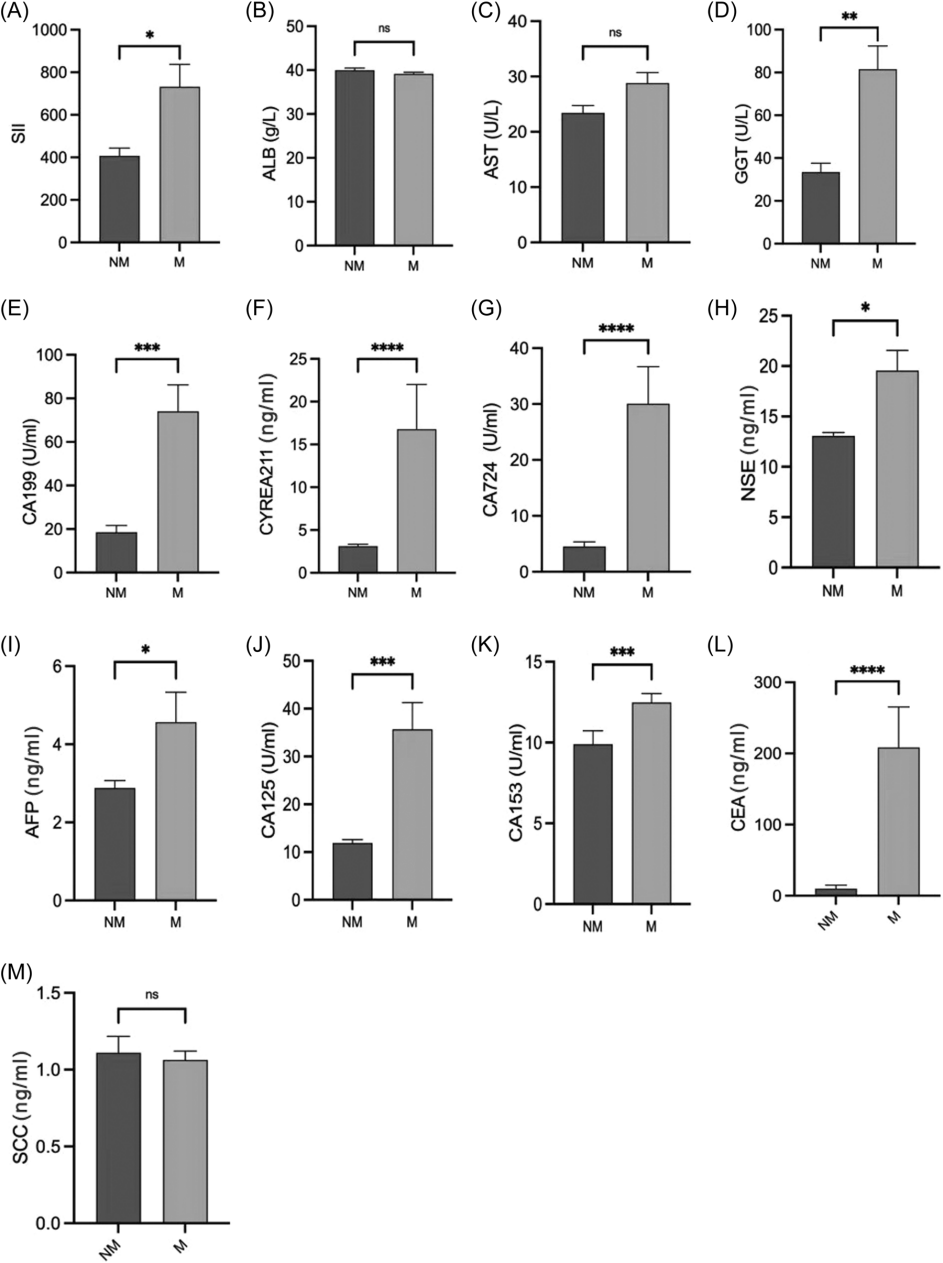
Differences in biomarkers between the metastasis group and the nonmetastasis group. *p < 0.05, **p < 0.01, ***p < 0.001, ****p < 0.0001. AFP, alpha fetoprotein; ALB, albumin; AST, aspartate aminotransferase; CA125, carbohydrate antigen 125; CA153, carbohydrate antigen 153; CA199, carbohydrate antigen 199; CA724, carbohydrate antigen 724; CEA, carcinoembryonic antigen; CYFRA211, cytokeratin fragment 19; GGT, gamma‐glutamyl transpeptidase; HC, healthy control; M, metastasis group; NM, nonmetastasis group; NSE, neuron‐specific enolase; SCC, squamous cell carcinoma antigen; SII, systemic immune‐inflammation index.

The levels of TAT, PIC, tPAIC, DD, FDP, PT, INR, TT, Fbg, SII, GGT, CA199, CYREA211, CA724, NSE, AFP, CA125, CA153, CEA were significantly higher in the M group than in the NM group (*p* < 0.001, *p* < 0.001, *p* < 0.01, *p* < 0.0001, *p* < 0.0001, *p* < 0.05, *p* < 0.05, *p* < 0.01, *p* < 0.0001, *p* < 0.05, *p* < 0.01, *p* < 0.001, *p* < 0.0001, *p* < 0.0001, *p* < 0.05, *p* < 0.05, *p* < 0.001, *p* < 0.001, and *p* < 0.0001, respectively; Figure 1B–H and 1J–K; Figure 2A and 2D–L).

### 3.2 Evaluation of all molecular markers for diagnostic metastasis

**Figure 3:**
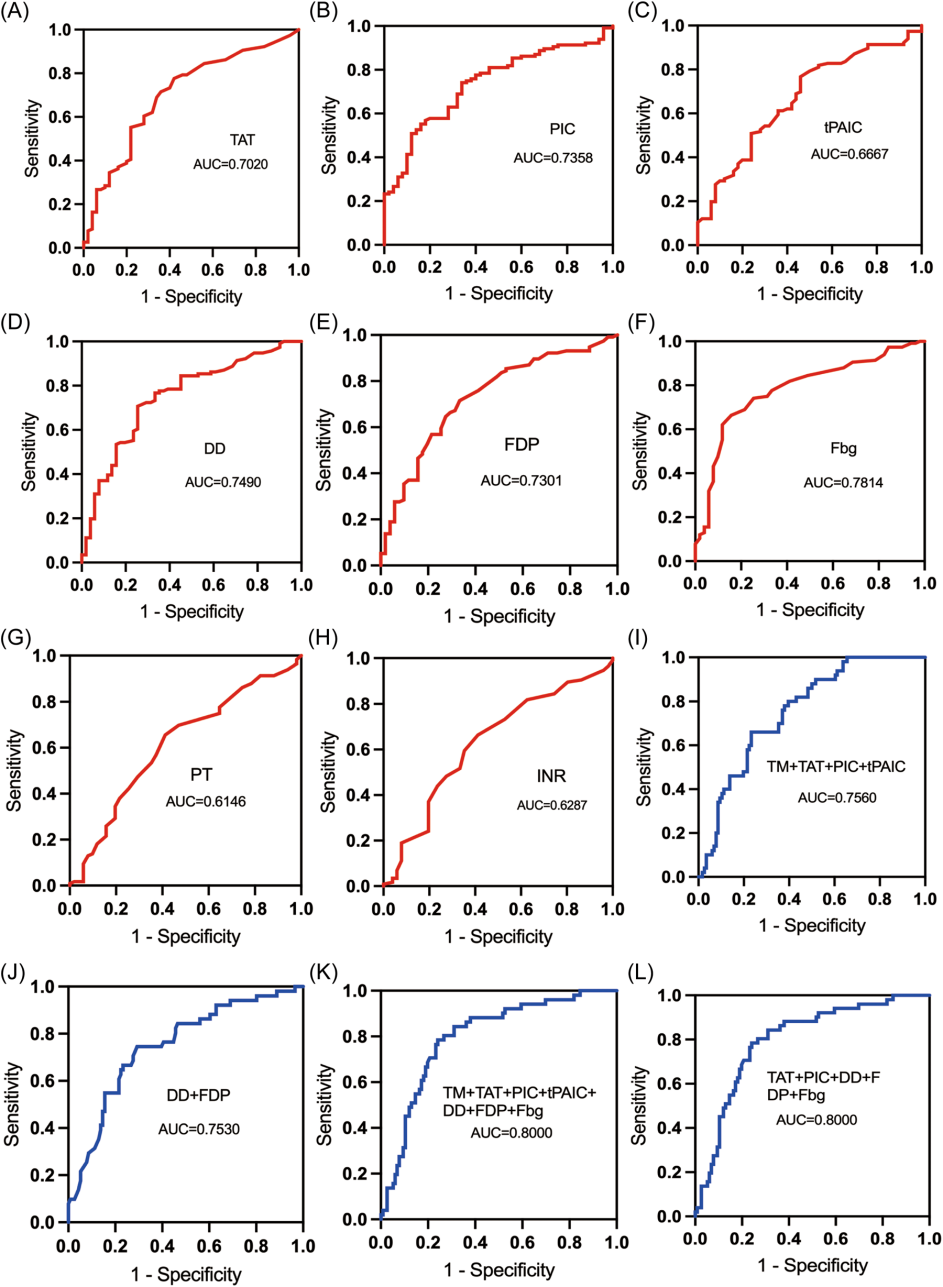
ROC curves of each marker and combined markers in the diagnosis of CRC metastasis. AUC, area under the curve; DD, d‐dimer; Fbg, fibrinogen; FDP, fibrin degradation product; INR: international normalized ratio; PIC, α2‐plasmininhibitor‐plasmin complex; PT, prothrombin time; ROC, the receiver operating characteristic curve; TAT, thrombin‐antithrombin complex; t‐PAIC: tissue plasminogen activator‐inhibitor complex.

The AUCs of coagulation markers TAT, PIC, tPAIC, DD, FDP, Fbg, PT, INR for diagnosing metastasis were 0.7020, 0.7358, 0.6667, 0.7490, 0.7301, 0.7814, 0.6146, 0.6287, respectively (Figure 3A–H). Their corresponding cutoff values were 1.35 μg/L, 0.678 mg/L, 6.1 μg/L, 0.535 mg/L, 2.25 mg/L, 3.25 g/L, 11.05 s, 0.955 (Table 2). PIC, DD, Fbg, and CEA showed a better performance for the diagnosis of metastasis, with sensitivities of 74.1%, 70.7%, 66.4%, and 66.7% and specificities of 66%, 76%, 84%, and 89.4%, respectively (Table 2). TM, TT, AT, and SII showed worse diagnostic efficiency (AUC = 0.5260, 0.3450, 0.5227, and 0.6009) (Table 2).

**Table 2:** Diagnostic efficacy of each molecular marker for the diagnosis of metastasis in patients with colorectal cancer.

Variable	AUC	Cutoff value	95% CI	*p* value	Sensitivity (%)	Specificity (%)	Youden index
TM	0.5260	9.05	0.4297–0.6224	0.60	50	62	0.12
TAT	0.7020	1.35	0.6150–0.7889	<0.001*	77.60	58	0.356
PIC	0.7358	0.678	0.6577–0.8139	<0.001*	74.10	66	0.401
tPAIC	0.6667	6.1	0.5781–0.7553	<0.001*	76.70	54	0.307
DD	0.7490	0.535	0.6684–0.8295	<0.001*	70.70	76	0.467
FDP	0.7301	2.25	0.6476–0.8125	<0.001*	71.60	68	0.396
Fbg	0.7814	3.25	0.7062–0.8567	<0.001*	66.40	84	0.504
PT	0.6146	11.05	0.5210–0.7082	0.02*	65.50	60	0.255
INR	0.6287	0.955	0.5356–0.7218	0.008*	66.40	60	0.264
APTT	0.5533	28.75	0.4623–0.6443	0.27	25.90	84	0.099
TT	0.3450	19.3	0.2530–0.4360	<0.001*	0.90	100	0.009
AT	0.5227	89.5	0.4251–0.6202	0.64	65.50	50	0.155
SII	0.6009	691.195	0.5116–0.6902	0.04*	29.8	91.5	0.213
CEA	0.7990	4.9	0.7318–0.8684	<0.001*	66.70	89.40	0.561
CA199	0.6828	11.26	0.5967–0.7688	<0.001*	76.30	55.30	0.316
CA125	0.6815	17.8	0.6017–0.7613	<0.001*	50.90	91.50	0.424

Abbreviations: APTT, activated partial thromboplastin time; AUC, area under the curve; CI, confidence interval; DD, d‐dimer; Fbg, fibrinogen; FDP, fibrin degradation product; INR, international normalized ratio; PT, prothrombin time; TAT, thrombin‐antithrombin complex; TM, thrombomodulin; t‐PAIC, tissue plasminogen activator‐inhibitor complex; TT, thrombin time.

* *p* < 0.05 considered statistically significant.

When a combination of seven coagulation markers (TM, TAT, PIC, tPAIC, DD, FDP, and Fbg) was used, the AUCs of “TM + TAT + PIC + tPAIC + DD + FDP + Fbg” and “TAT + PIC + DD + FDP + Fbg” were higher than the AUCs of “TM + TAT + PIC+tPAIC” and “DD + FDP” for the diagnosis of metastasis in CRC patients. The AUC, sensitivity, and specificity of “TM + TAT + PIC + tPAIC” were 0.7560, 76.7%, and 64%, respectively. The AUC, sensitivity, and specificity of “DD + FDP” were 0.7530, 70.7%, and 76%, respectively. The AUC, sensitivity, and specificity of “TAT + PIC + DD + FDP + Fbg” were 0.8000, 76.7%, and 78%, respectively, with the same result as that for “TM + TAT + PIC + tPAIC + DD + FDP + Fbg.” Therefore, the combined detection of TAT, PIC, DD, FDP, and Fbg showed a better diagnostic efficacy for the identification of metastasis in CRC patients (Table 3 and Figure 3I–L).

**Table 3:** Combined diagnostic markers of metastasis by ROC curve.

Variable	AUC	95% CI	*p* value	Sensitivity (%)	Specificity (%)	Youden index
TM + TAT + PIC + tPAIC	0.7560	0.682–0.830	<0.001*	76.70	64	0.427
TM + TAT + PIC + tPAIC+DD + FDP + Fbg	0.8000	0.729–0.872	<0.001*	76.70	78	0.547
DD + FDP	0.7530	0.672–0.834	<0.001*	70.70	76	0.467
TAT + PIC + DD + FDP + Fbg	0.8000	0.729–0.872	<0.001*	76.70	78	0.547

Abbreviations: AUC, area under the curve; CI, confidence interval; DD, d‐dimer; Fbg, fibrinogen; FDP, fibrin degradation product; PIC, α2‐plasmininhibitor‐plasmin complex; ROC, receiver operating characteristic; TAT, thrombin‐antithrombin complex; TM, thrombomodulin.

* *p* < 0.05 considered statistically significant.

### 3.3 Evaluation of the correlation between TM, TAT, PIC, tPAIC, DD, and FDP and prognosis

**Figure 4:**
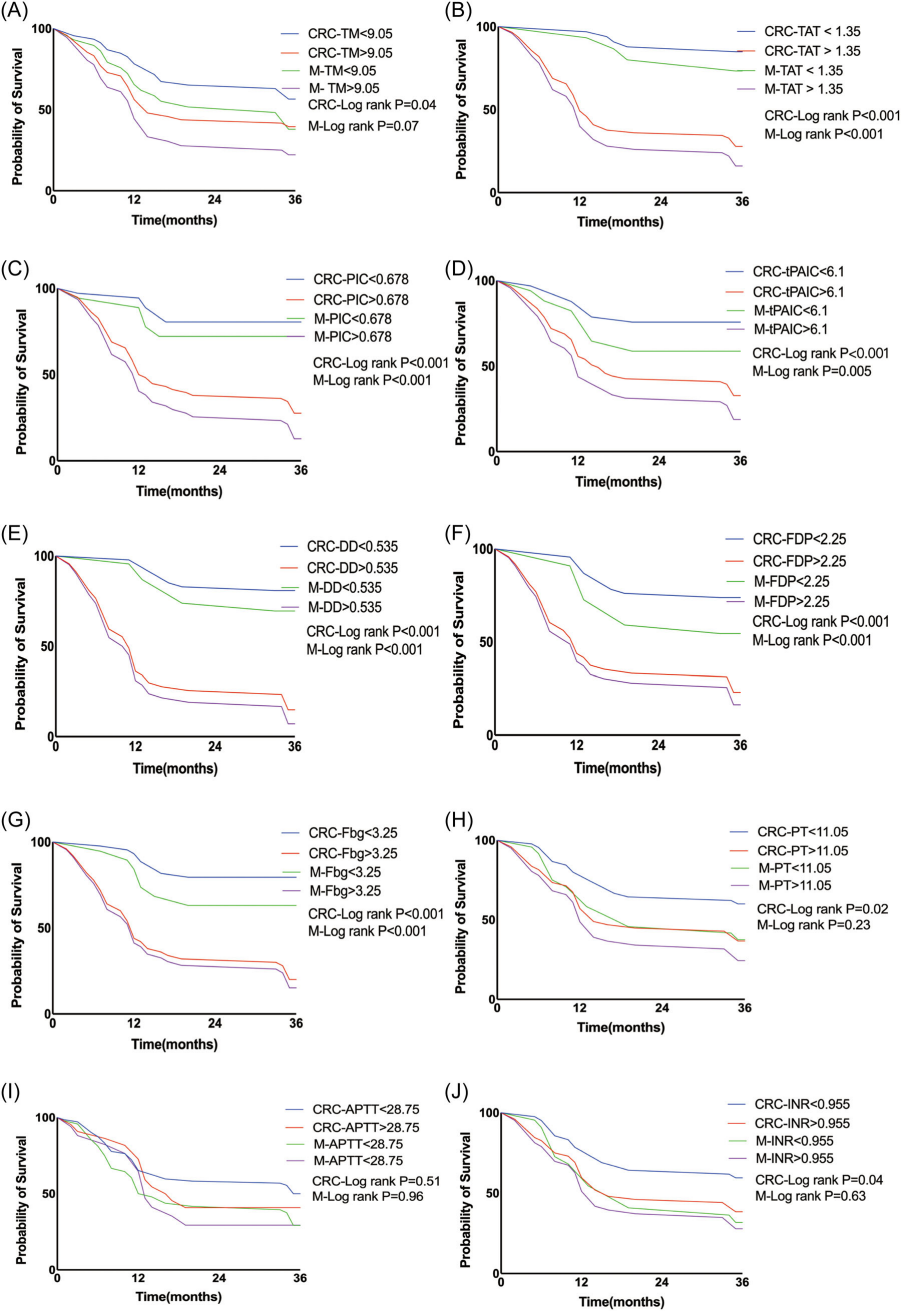
Kaplan‒Meier survival curves of TM, TAT, PIC, tPAIC, DD, FDP, Fbg, PT, INR, and APTT and overall survival in CRC and in the M group. APTT, activated partial thromboplastin time; CRC, colorectal cancer group; DD, d‐dimer; Fbg, fibrinogen; FDP, fibrin degradation product; INR, international normalized ratio; M, metastasis group; PIC, α2‐plasmininhibitor‐plasmin complex; PT, prothrombin time; TAT, thrombin‐antithrombin complex; TM, thrombomodulin; t‐PAIC, tissue plasminogen activator‐inhibitor complex.

To assess the function of the coagulation indicators TM, TAT, PIC, tPAIC, DD, FDP, Fbg, PT, INR, and APTT in CRC, survival studies were carried out. The cutoff values for TM, TAT, PIC, tPAIC, DD, FDP, Fbg, PT, INR, and APTT were 9.05 TU/mL, 1.35 μg/L, 0.678 mg/L, 6.1 μg/L, 0.535 mg/L, 2.25 mg/L, 3.25 g/L, 11.05 s, 0.955 s, and 28.75 s, respectively. Patients were categorized as the high‐level group if their marker levels above the threshold limits (TM > 9.05 TU/mL, TAT > 1.35 μg/L, PIC > 0.678 mg/L, tPAIC >6.1 μg/L, DD > 0.535 mg/L, FDP > 2.25 mg/L, Fbg > 3.25 g/L, PT > 11.05 s, INR > 0.955, and APTT > 28.75 s), whereas low‐level groups were those with lower levels. The high‐level groups of TM, TAT, PIC, tPAIC, DD, FDP, Fbg, PT, and INR had considerably shorter OS than the low‐level group, according to the results of the Kaplan–Meier survival analysis (CRC‐log rank *p* = 0.04, *p* < 0.001, *p* < 0.001, *p* < 0.001, *p* < 0.001, *p* < 0.001, *p* < 0.001, *p* = 0.02, and *p* = 0.04, respectively, Figure 4A–H and 4J). However, no such association was observed for APTT in CRC (CRC‐log rank, *p* = 0.51, Figure 4I). The prognosis of patients with CRC in the metastatic group was then evaluated in connection to TM, TAT, PIC, tPAIC, DD, FDP, Fbg, PT, INR, and APTT. The results showed that TAT, PIC, tPAIC, DD, FDP, and Fbg can be used as prognostic indicators in CRC with metastasis (M‐log rank *p* < 0.001, *p* < 0.001, *p* = 0.005, *p* < 0.001, *p* < 0.001, and *p* < 0.001, respectively, Figure 4B–G). However, TM, PT, APTT, and INR could not be used as prognostic indicators in M (M‐log rank *p* = 0.07, *p* = 0.23, *p* = 0.96, *p* = 0.63, respectively, Figure 4A and 4H–J).

### 3.4 Independent OS prognotic factors

To find independent predictive indicators for OS in CRC patients, Cox regression analysis were carried out (Table 4). Single‐variate analysis revealed that TM (hazard ratio [HR] = 2.556, *p* = 0.01), DD (HR = 5.971, *p* = 0.02), CA125 (HR = 3.691, *p* < 0.001), and CEA (HR = 7.13, *p* < 0.001) were OS predictors with a poor prognosis. The multivariate analysis found that TM (HR = 2.226, *p* = 0.02), DD (HR = 4.376, *p* < 0.001), CA125 (HR = 3.851, *p* < 0.001), and CEA (HR = 5.929, *p* < 0.001) remain independent prognostic factors. The coagulation indicators TM and DD have been recognized as independent prognostic factors.

**Table 4:** Predictors of overall survival.

Cox for OS variable	Univariate analysis		Multivariate analysis	
	HR (95% CI)	*p* value	HR (95% CI)	*p* value
Sex (male vs. female)	1.212 (0.584–2.515)	0.61		
Age (<64 vs. ≥64 years)	0.341 (0.156–0.748)	0.007*	0.433 (0.227–0.825)	0.01*
Hypertension	1.744 (0.721–4.22)	0.22		
Diabets	1.311 (0.546–3.15)	0.55		
TM (<9.05 vs. ≥9.05)	2.556 (1.211–5.396)	0.01*	2.226 (1.145–4.327)	0.02*
TAT (<1.35 vs. ≥1.35)	0.73 (0.209–2.552)	0.62		
PIC (<0.678 vs. ≥0.678)	2.345 (0.774–7.105)	0.13		
tPAIC (<6.1 vs. ≥6.1)	1.075 (0.39–2.962)	0.89		
DD (<0.535 vs. ≥0.535)	5.971 (1.373–25.963)	0.02*	4.376 (1.932–9.914)	<0.001*
FDP (<2.25 vs. ≥2.25)	0.572 (0.17–1.924)	0.37		
Fbg (<3.25 vs. ≥3.25)	1.044 (0.409–2.665)	0.93		
SII (<691.195 vs. ≥691.195)	1.151 (0.548–2.419)	0.71		
CA125 (<17.8 vs. ≥17.8)	3.691 (1.706–7.983)	<0.001*	3.851 (1.961–7.562)	<0.001*
CEA (<4.9 vs. ≥4.9)	7.13 (2.46–20.663)	<0.001*	5.929 (2.447–14.368)	<0.001*
CA199 (<11.26 vs. ≥11.26)	0.361 (0.111–1.176)	0.09		

Abbreviations: CI, confidence interval; DD, d‐dimer; Fbg, fibrinogen; FDP, fibrin degradation product; HR, hazard ratio; OS, overall survival; PIC, α2‐plasmininhibitor‐plasmin complex; TAT, thrombin‐antithrombin complex; TM, thrombomodulin; t‐PAIC, tissue plasminogen activator‐inhibitor complex.

* *p* < 0.05 considered statistically significant.

### 3.5 Formulation of the prognostic nomogram

**Figure 5:**
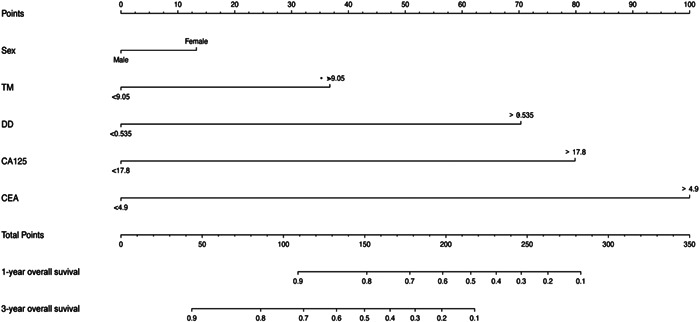
Nomogram integrating sex, TM, DD, CA125, and CEA for 1‐ and 3‐year overall survival in CRC. CA125, carbohydrate antigen 125; CEA, carcinoembryonic antigen; CRC, colorectal cancer; DD, d‐dimer; TM, thrombomodulin.

The independent prognostic indicators discovered in the multivariate Cox regression analysis were included in a prognostic nomogram that was created to predict 1‐ and 3‐year OS. These included sex, TM, DD, CA125, and CEA (Figure 5). By projecting to the top “points” axis in accordance with the patient's circumstances, it is possible to determine the equivalent score for each variable. The total points are determined in a similar manner by adding the matching scores for all variables. The 1‐ and 3‐year OS can be estimated by projecting the total points to the bottom “1‐year overall survival” and “3‐year overall survival” axes.

## 4 DISCUSSION

The mechanism underlying the development of the hypercoagulable state in malignant tumors remains unclear. However, increasing numbers of studies indicate that the hypercoagulability of blood is closely associated with cancer growth, progression, and metastasis.^23^, ^24^ The most frequent causes of death in cancer patients are thrombotic and/or hemorrhagic complications.^25^ Thrombosis is a multifactorial process related to coagulation, fibrinolysis, and the endothelial system,^26^, ^27^ which are factors related to the development of tumors. In addition, coagulation process is closely related to tumor metastasis.^28^ Tissue factor (TF), which is expressed on the surface of tumor cells, on microparticles, or on the tumor stroma, activates the extrinsic route and causes the synthesis of fibrin. Selectins interact with their ligands, which are expressed on tumor cells, to recruit platelets. Platelet aggregation is also brought on by the COX enzyme expressed on tumor cells and the endothelium, which generates prostaglandins and thromboxanes. Therefore, thrombin affects tumor metastasis through two independent mechanisms.^29^, ^30^

The blood coagulation and fibrinolysis mechanisms in the human body operate in dynamic equilibrium under normal conditions. TAT, a complex created by the interaction of thrombin and antithrombin, is a reliable indicator of the body's thrombin levels. Elevated TAT indicates coagulation activation, which exists in cancer patients.^31^ Yang et al.^32^ showed that patients with malignant tumors might use elevated plasma TAT levels to diagnose thrombosis because they signify an aberrant increase in coagulation and fibrinolytic activity.

TM, a thrombin‐regulating protein, is not only a marker of endothelial damage, but also forecast the state and alterations in the body's coagulation function.^19^ Binding of thrombin to TM changes its function from a component that promotes clotting to a powerful anticoagulant, activating protein C, which in turn inactivates factors Va and VIIIa together with its cofactor protein S, thereby downregulating thrombin production over the endothelial cell surface and anticoagulation.^33^

In this study, the levels of TM and TAT were significantly higher in patients with CRC than in the HC group (*p* < 0.001). The level of TAT was significantly higher in the metastasis group than in the nonmetastasis group (*p* < 0.001), whereas TM did not differ between the two groups. The results showed that the endothelium of patients with CRC is damaged, resulting in the release of TM into the blood and the promotion of coagulation.

Fibrinolysis is activated when the coagulation system is activated. Thrombin released by vascular endothelial cells can cause passive activation of the fibrinolytic system. Cross‐linked fibrin hydrolysis and secondary fibrinolysis to form DD and FDP reflect the course of thrombosis in patients with CRC.^34^, ^35^ In this study, we showed that DD and FDP were higher in patients with CRC than in HCs and higher in the M group than in the NM group. There was a significant statistical difference, which confirmed this process. We also showed that the levels of PIC and tPAIC were significantly higher in CRC than in healthy individuals and higher in the M group than in the NM group. This suggests that in the process of fibrinolysis, fibrinolytic activity decreases, and the balance of coagulation and fibrinolysis shifts in the direction of thrombosis, confirming that the increase in molecular markers of thrombosis is closely related to the occurrence of CRC metastasis.

Zhou et al.^36^ reported that TM, TAT, PIC, and t‐PAIC are effective diagnostic and prognostic markers of venous thromboembolism in cancer patients, and that a combination of the four markers shows better efficacy than a single marker. Consistent with previous studies, TAT, PIC, DD, FDP, and Fbg were useful markers for the diagnosis of metastasis in CRC patients, according to the results of ROC analysis. ROC curve analysis showed that the AUCs of TAT, PIC, DD, FDP, and Fbg for predicting metastasis in CRC were 0.7020, 0.7358, 0.7490, 0.7301, and 0.7814. And each of these indices differed significantly from the others. The predictive sensitivities of TAT, PIC, t‐PAIC, DD, and FDP were 77.6%, 74.1%, 76.7%, 70.7%, and 71.6%, respectively, indicating that plasma TAT has a higher predictive value for metastasis of CRC than the other indicators. In the diagnosis of metastasis, the combination of TAT, PIC, DD, FDP, and Fbg showed the highest AUC (0.8000) and better sensitivity (76.7%) and specificity (78%), which were superior to those of a single marker. Thus, TAT and PIC combined with DD, FDP, and Fbg can be used as specific markers for the diagnosis of metastasis in CRC patients. High levels of TM, DD, Fbg, FDP, PIC, TAT, and tPAIC indicated poorer OS. Multivariate Cox regression analysis also showed that TM (HR = 2.226, *p* = 0.02), DD (HR = 4.376, *p* < 0.001), CA125 (HR = 3.851, *p* < 0.001), and CEA (HR = 5.929, *p* < 0.001) were independent prognostic variables for CRC patients. Although, TAT, PIC, and t‐PAIC cannot be utilized as predictive markers, they can be employed as sensitive markers for the diagnosis of metastasis.

## 5 CONCLUSION

In conclusion, coagulation indicators are useful for determining a patient's prognosis for CRC. TM and DD were found to be independent predictors of OS. A nomogram integrating TM, DD, CA125, and CEA was established to forecast CRC patients’ prognosis.

## AUTHOR CONTRIBUTIONS

**Wenxin Chen**: Formal analysis; Writing—original draft; Writing—review & editing. **Yueying Li**: Formal analysis; Resources; Software. **Weifeng Wang**: Data curation; Funding acquisition; Resources. **Yingjun Xue**: Resources. **Jianxin Qian**: Supervision. **Weiwei Liu**: Funding acquisition; Project administration; Supervision. **Xiaobo Hu**: Conceptualization; Project administration; Supervision.

## CONFLICT OF INTEREST STATEMENT

The authors declare no conflicts of interest.

## TRANSPARENCY STATEMENT

The lead author Jianxin Qian, Weiwei Liu, Xiaobo Hu affirms that this manuscript is an honest, accurate, and transparent account of the study being reported; that no important aspects of the study have been omitted; and that any discrepancies from the study as planned (and, if relevant, registered) have been explained.

## Data Availability

The findings of this study are available from the corresponding author upon reasonable request.
